# Assessment of Different Castration Resistance Definitions and Staging Modalities in Metastatic Castration-Resistant Prostate Cancer

**DOI:** 10.3390/cancers16203506

**Published:** 2024-10-17

**Authors:** Mike Wenzel, Benedikt Hoeh, Clara Humke, Carolin Siech, Cristina Cano Garcia, Georg Salomon, Tobias Maurer, Markus Graefen, Simon Bernatz, Andreas Michael Bucher, Luis Kluth, Felix K. H. Chun, Philipp Mandel

**Affiliations:** 1 Department of Urology, University Hospital Frankfurt, Goethe University Frankfurt am Main, 60629 Frankfurt, Germany; 2 Martini-Klinik Prostate Cancer Center, University Hospital Hamburg-Eppendorf, 20251 Hamburg, Germany; 3 Department of Diagnostic and Interventional Radiology, University Hospital Frankfurt, 60629 Frankfurt, Germany

**Keywords:** mCRPC, PSMA, PET, PFS, survival

## Abstract

**Background/Objectives**: Progression to metastatic castration-resistant prostate cancer (mCRPC) is defined either biochemically, radiographically or both. Moreover, staging for mCRPC can be performed either conventionally or with molecular imaging such as prostate-specific membrane antigen computer tomography (PSMA-PET/CT). **Methods**: We relied on the Frankfurt Metastatic Cancer Database of the Prostate (FRAMCAP) database to compare progression-free (PFS) and overall survival (OS) outcomes regarding the cause of castration resistance and the staging modality used. **Results**: Overall, 35% progressed to mCRPC biochemically vs. 23% radiographically vs. 42% biochemically + radiographically. The PSA nadir in mHSPC (1.4 vs. 0.4 vs. 0.8 ng/mL) and PSA level at mCRPC progression (15 vs. 2 vs. 21 ng/mL, both *p* ≤ 0.01) were significantly higher for biochemical vs. radiographic vs. both progressed patients. In PFS and OS analyses, no significant differences were observed among all three compared groups. In the comparison of the staging used for progression to mCRPC, 67% received conventional vs. 33% PSMA-PET/CT, with higher metastatic burden in mHSPC and osseous lesions in mCRPC for conventionally staged patients (both *p* < 0.01). In PFS (15.3 vs. 10.1 months, hazard ratio [HR]: 0.75) and OS analyses (52.6 vs. 34.3 months, HR: 0.61, both *p* < 0.05), PSMA-PET/CT harbored better prognosis; however, this did not hold after multivariable adjustment. Similar results were observed for further analyses in second- and third-line mCRPC or patients with a PSA level of ≥2 ng/mL. **Conclusions**: The cause of progression to mCRPC seems not to influence cancer-control outcomes, despite important baseline tumor characteristic differences. The PSMA-PET/CT staging modality might be associated with better PFS and OS outcomes, possibly due to its more sensitive detection of progression or new metastatic lesions.

## Simple Summary

The current real-world study suggests that important differences exist in patients progressing to mCRPC. Specifically, we found that a proportion of over 20% of patients progress without a PSA level rise, emphasizing the importance of regular staging intervals. Moreover, we found that patients with worse PSA responses are at higher risk for biochemical progression, emphasizing the need for regular PSA check-ups and kinetic measurements. However, no differences in PFS and OS outcomes between these patients were observed. Finally, staging with PSMA-PET/CT might provide better PFS and OS, relative to conventionally staged mCRPC patients. However, this observation may be substantially influenced by the detection of earlier disease progression and the possibility of treatment in patients with a lower metastatic burden, as well as more contemporary patients with better treatment options.

## 1. Introduction

Prostate cancer is the second most common cancer in men worldwide and was diagnosed 1.4 million times in 2020 [1]. A newly diagnosed metastatic prostatic disease in the first-line treatment stage is called metastatic hormone-sensitive prostate cancer (mHSPC), while after progression on first-line treatment, it is called metastatic castration-resistant prostate cancer (mCRPC). mCRPC remains the fatal advanced stage of prostate cancer after its initial hormone-sensitive status, despite the approval of several life-prolonging agents for mCRPC within recent years [2,3,4,5,6,7,8,9]. Therefore, clinicians aim to maximally extend the time until progression from mHSPC to mCRPC with systemic treatment [7,8,9]. The current definition of mCRPC was firstly introduced in 2008 by the Prostate Cancer Working Group [10,11,12]. The current European Urology Association (EAU) biochemical definition of mCRPC is a rise in the prostate-specific antigen (PSA) level above 2 ng/mL and at least 50% above the treatment nadir years [13]. Moreover, radiographic progression to mCRPC is defined as an occurrence of at least two new bone metastases or one new soft tissue metastasis (on conventional imaging) regarding the Response Evaluation Criteria in Solid Tumors (RECIST) criteria [14]. Both criteria presume a sufficiently suppressed testosterone level of <50 ng/mL. One may assume that patients with radiographic progression may harbor worse cancer-control outcomes than patients with only a biochemical PSA level progression.

However, with advances in prostate cancer treatment for mHSPC and mCRPC, novel combination therapies in addition to androgen deprivation therapy (ADT) are currently routinely used, achieving substantially lower PSA nadir values and more sufficient testosterone suppression compared to the historically used ADT monotherapy [15,16,17,18]. Moreover, additional technological advances in staging modalities such as prostate-specific membrane antigen positron emission computer tomography (PSMA-PET/CT) have shown superiority regarding accuracy, and sensitivity in initially diagnosed prostate cancer patients when compared to conventional staging modalities such as CT or bone scans [19]. However, data on cancer-control outcomes regarding the cause of progression to mCRPC and the staging modalities used are currently under investigation and scant [20].

We addressed these knowledge gaps and relied on the FRAMCAP (Frankfurt Metastatic Cancer Database of the Prostate) to investigate cancer-control outcomes such as PFS and OS in mCRPC patients stratified by cause of progression to mCRPC: biochemically, radiographically or both. Moreover, we additionally aimed to investigate PFS and OS outcomes regarding the staging modality used in progression to mCRPC and beyond. We hypothesized that substantial differences exist between mCRPC patients with biochemical vs. radiographic progression, as well as patients staged with PSMA-PET/CT or conventionally.

## 2. Materials and Methods

### 2.1. Study Population

With approval from the local ethics committee (reference number: SUG-5-2024) and in compliance with the principles of the Declaration of Helsinki, we conducted a retrospective identification of all metastatic prostate cancer patients from the prospectively sampled FRAMCAP database. All patients (*n* = 1164) received treatment at the Department of Urology, University Hospital Frankfurt, Germany. For the analysis, only patients who had progressed to castration-resistant prostate cancer (mCRPC) were included. In the first part of the analyses, patients were excluded if the reason for mCRPC progression was unknown, resulting in 356 mCRPC patients. In the second part of the analyses, patients with unknown staging modality at the time of progression to mCRPC were excluded, resulting in 341 eligible mCRPC patients.

### 2.2. mCRPC Definition

mCRPC was defined according to the EAU guidelines [13]: Three consecutive rises in PSA values during mHSPC treatment with a PSA level above 2 ng/mL and a 50% rise above the nadir were considered as biochemical progression. Moreover, two new osseous or one soft tissue metastasis using the RECIST 1.1 criteria was considered as mCRPC radiographic progression.

### 2.3. Staging Modalities

Patients staged with either CT or magnetic resonance imaging (MRI) and/or bone scan were categorized as having received conventional imaging. Patients receiving both conventional and PSMA-PET/CT imaging were excluded (*n* = 3).

### 2.4. Statistical Analysis

The descriptive statistics included frequencies and proportions for categorical variables. For continuous variables, median values and interquartile ranges (IQRs) were reported. The Chi-square test was used to evaluate the statistical significance of differences in proportions, while the *t*-test and Kruskal–Wallis test were employed to analyze differences in distributions.

In the first set of PFS and OS analyses, patients were stratified according to the reason for mCRPC progression: biochemical vs. radiographic vs. both. In the second part of the PFS and OS analyses, patients were stratified according to the conventional vs. PSMA-PET/CT staging modality. Sensitivity analyses separately addressed second- and third-line mCRPC treatments. To reduce the lead time and selection bias, sensitivity analyses were performed for patients with a PSA level of ≥2 ng/mL, when the biochemical cut-off for castration resistance was achieved, as some patients might have undergone PSMA-PET/CT earlier.

For all cancer-control outcome analyses, univariable and multivariable Cox regression models were applied. Adjustments to the multivariable Cox regression models were performed for mCRPC progression analyses for the age of mHSPC patients, Eastern Cooperative Oncology Group (ECOG) status, high-volume mHSPC, de novo mHSPC, cardiovascular disease, Gleason Score and mCRPC treatment and additionally for staging comparisons for the PSA level at mCRPC progression and the year of treatment. OS analyses were additionally adjusted for the number of received systemic treatment lines. All tests were two-sided, with the level of significance set at *p* < 0.05. The R software environment for statistical computing and graphics (version 4.0.0, R Foundation for Statistical Computing, Vienna, Austria) was used for all analyses.

## 3. Results

### 3.1. Baseline Characteristics: PSA vs. Radiographic Progression

In a comparison of 356 mHSPC patients who progressed to mCRPC, 35% (*n* = 126) progressed biochemically vs. 23% (*n* = 81) radiographically vs. 42% (*n* = 149) due to both causes (Table 1). The median age at mHSPC diagnosis and PSA level in mHSPC were 69 vs. 69 vs. 67 years and 84 vs. 34 vs. 64 ng/mL, respectively, for the groups of biochemical vs. radiographic vs. both progression causes (both *p* ≥ 0.2). The proportions with ECOG ≥ 2 ranged from 3.4 to 6.0% (*p* = 0.5).

Significant differences among the three compared groups were observed for the absolute PSA nadir during mHSPC treatment for biochemical vs. radiographic vs. both causes (1.4 vs. 0.4 vs. 0.8 ng/mL, *p* = 0.01). Moreover, the PSA level at mCRPC progression was lowest for radiographic patients compared to biochemical and both causes (2 vs. 15 vs. 21 ng/mL, *p* < 0.001). However, no differences in the rates of ≥90% PSA responses were observed among all three groups; the rates ranged between 78% and 85% (*p* = 0.3). Moreover, patients with PSA progression had significantly higher proportions of de novo mHSPC (74% vs. 54% vs. 64%, *p* = 0.01) and numerically higher rates of high-volume mHSPC metastatic burden (67% vs. 51% vs. 50%, *p* = 0.07) relative to mHSPC with radiographic progression or progression due to both causes. Additionally, in mCRPC, the rates of visceral metastases were higher for the radiographic cohort compared to the PSA and both-causes cohort (22% vs. 8.6% vs. 9.8%, *p* = 0.2). No significant or clinically meaningful differences were observed in comparisons between mHSPC and mCRPC treatments; however, the distribution of received numbers of systemic treatment lines differed significantly (Table 2).

### 3.2. Oncological Outcomes: PSA vs. Radiographic Progression

**Figure 1:**
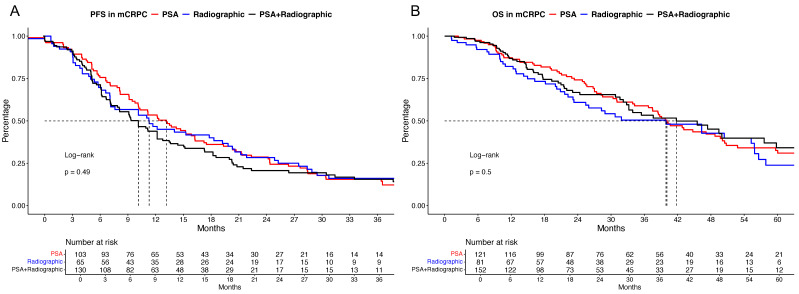
Kaplan–Meier curves depicting progression-free survival (PFS, (**A**)) and overall survival (OS, (**B**)) in metastatic castration-resistant prostate cancer (mCRPC) patients stratified according to biochemical (PSA) vs. radiographic vs. both (PSA + radiographic) reasons for initial progression to castration resistance.

In cancer-control outcome measurements, no significant difference in PFS was observed for mHSPC patients who progressed biochemically vs. radiographically vs. due to both causes (Figure 1A), with median PFS times of 13.2 vs. 11.3 vs. 10.1 months (*p* = 0.49) and 24-month PFS rates of 28.7% vs. 28.4% vs. 20.7%. After further multivariable adjustment for potential confounding variables in the Cox regression models, no significant PFS differences were observed.

In OS analyses (Figure 1B), no significant differences among the three compared groups were observed, with median OS times of 40.1 vs. 39.9 vs. 41.8 months for biochemical vs. radiographic vs. biochemical and radiographic progression (*p* = 0.5), with 48-month OS rates of 42.3% vs. 42.7% vs. 45.2%. After multivariable adjustment, no significant OS differences were observed.

### 3.3. Baseline Characteristics: Staging at Progression to mCRPC

In a comparison of 352 mCRPC patients (Table 3), 67% (*n* = 235) received conventional and 33% (*n* = 117) received PSMA-PET/CT staging. The median PSA level in mHSPC was 75 vs. 54 ng/mL for conventional vs. PSMA-PET/CT staging (*p* = 0.11). No significant differences were observed for the PSA nadir in mHSPC (0.8 vs. 1.0 ng/mL) or the proportion of ≥90% PSA responses (87 vs. 83%) for conventional vs. PSMA-PET/CT staging (both ≥0.5). Numerically, the PSA level in mCRPC was higher in conventionally staged patients (19 vs. 10 ng/mL) vs. PSMA-PET/CT patients (*p* = 0.07).

Significant differences were observed regarding higher proportions with ECOG status ≥ 2 (9.6 vs. 2.3%) and de novo (60% vs. 48%) and high-volume mHSPC (64 vs. 41%, all *p* ≤ 0.01) in patients who received conventional imaging vs. PSMA-PET/CT. Conversely, the rates of local therapies were higher in the PSMA-PET/CT cohort (56 vs. 40%, *p* = 0.028). Moreover, the rates of M1a disease in mCRPC were significantly higher for PSMA-PET/CT-staged patients (19% vs. 6.2%, *p* < 0.01). No significant difference in the rates of the cause of progression to mCRPC was observed, with numerically higher rates of radiographic progression in PSMA-PET/CT patients (32% vs. 23%, *p* = 0.1). Regarding the treatment distribution for mHSPC and mCRPC, significant differences were observed (both *p* ≤ 0.01) and are displayed in Table 4.

### 3.4. Oncological Outcomes: Staging at Progression to mCRPC

**Figure 2:**
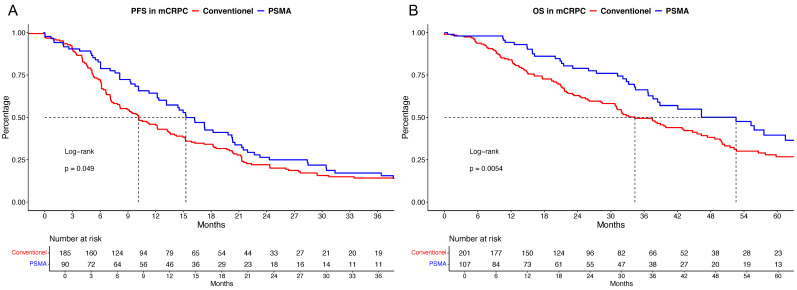
Kaplan–Meier curves depicting progression-free survival (PFS, (**A**)) and overall survival (OS, (**B**)) in metastatic castration-resistant prostate cancer (mCRPC) patients stratified according to conventional vs. prostate-specific membrane antigen positron emission computer tomography (PSMA) staging.

In PFS analyses (Figure 2A), a significant advantage for PSMA-PET/CT relative to conventional staging was observed, with median PFS times of 15.3 vs. 10.1 months (hazard ratio [HR]: 0.75, *p* = 0.049) and 24-month PFS rates of 26.4% vs. 22.1%. However, after multivariable adjustment in the Cox regression models, no significant difference remained (*p* = 0.3).

In OS analyses (Figure 2B), significant differences were also observed, with median OS times of 52.6 vs. 34.3 months for PSA-PET/CT vs. conventional imaging (HR: 0.61, *p* < 0.01) and 48-month OS rates of 50.1% vs. 38.3%. However, after additional adjustment, no OS difference was found (*p* = 0.8).

**Figure 3:**
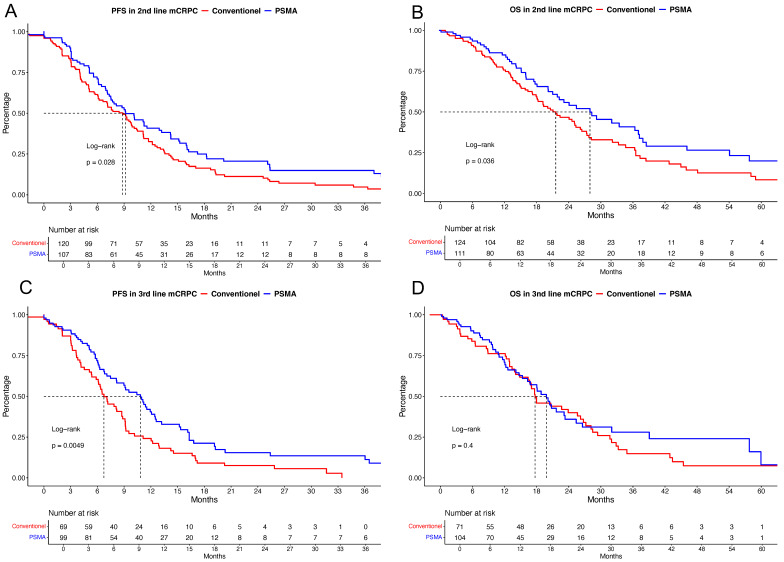
Kaplan–Meier curves depicting progression-free survival (PFS, (**A**)) and overall survival (OS, (**B**)) in second-line metastatic castration-resistant prostate cancer (mCRPC), as well as PFS (**C**) and OS (**D**) in third-line mCRPC patients, stratified according to conventional vs. prostate-specific membrane antigen positron emission computer tomography (PSMA) staging.

In further analyses of second- and third-line mCRPC patients (Figure 3), significant advantages were observed for PSMA-PET/CT staging vs. conventional staging regarding PFS and OS in second-line mCRPC and PFS in third-line mCRPC (all *p* < 0.036); however, no differences in OS from third-line mCRPC onwards were observed (*p* = 0.4). Multivariable analyses showed no differences regarding PFS and OS outcomes.

Sensitivity analyses of patients with a PSA level of ≥2 ng/mL revealed similar results to those in the overall cohort, with median PFS times of 16.2 vs. 10.1 months (*p* = 0.067) and OS times of 46.3 vs. 37.5 months (*p* = 0.028) for PSMA-PET/CT vs. conventional staging, respectively. The corresponding 24-month PFS and 48-month OS rates were 48.4% vs. 37.4% and 27.2% vs. 20.9% for PSMA-PET/CT vs. conventional staging. Multivariable adjusted analyses also showed no differences regarding PFS and OS outcomes.

## 4. Discussion

We initially hypothesized that important baseline and cancer-control outcome differences exist for mHSPC patients progressing to mCRPC regarding the cause of progression. Moreover, we hypothesized that the staging modality used for mCRPC-progressing patients could also influence cancer-control outcomes. We tested these hypotheses within the FRAMCAP database and arrived at several important observations.

First, we observed that the majority of mHSPC patients progressed to castration resistance based on combined biochemical and radiographic progression (42%), while the lowest proportion of mHSPC patients progressed only radiographically (23%), and approximately one-third progressed with a rise in their PSA level (35%, Table 1). Comparing the baseline patient and tumor characteristics of these three groups, we found that patients progressing due to a PSA rise harbored significantly or clinically meaningful higher rates of de novo mHSPC and high-volume mHSPC, as well as higher PSA nadirs in mHSPC and PSA levels at mCRPC progression, relative to both compared groups. These observations are important since they emphasize that patients with a high metastatic burden at metastatic occurrence are at the highest risk of (early) biochemical progression and should therefore be closely monitored by PSA testing. This recommendation is emphasized by the previous findings of two other studies, showing that de novo high-volume mHSPC patients usually harbor the worst oncological outcomes and, therefore, most likely need closer monitoring [21]. Additionally, despite regular PSA testing in follow-ups, PSA nadir information should be sampled in this patient cohort, and early progression may be suspected if the PSA does not fall below the definition of an undetectable PSA level (historical cut-off: <0.2 ng/mL, new cut-off: ≤0.02 ng/mL) [15,17,18]. Conversely, monitoring should not only be based on PSA testing, since within our cohort, 23% of the included patients progressed radiographically without reaching the biochemical definition of mCRPC. These observations are in agreement with previous findings. For example, a post hoc analysis of the prospective ENZAMET trial also found a rate of 10% of mHSPC patients progressing radiographically without a PSA rise [22]. This observation clearly emphasizes the need for regular radiographic staging intervals, even though no PSA rises occurred during mHSPC treatment.

Comparing cancer-control outcomes, no significant differences in PFS and OS outcomes were observed among the three groups. However, numerically, patients progressing based on PSA harbored only slightly better PFS than the other two compared groups, and the worst PFS was observed for progression due to PSA and radiographic causes (13.2 vs. 11.3 vs. 10.1 months, Figure 1). In a previously published real-world cohort, as well as the post hoc analyses of the ENZAMET trial, patients progressing radiographically without a PSA rise harbored the worst oncological outcomes [22,23,24]. This observation may be based on the occurrence of more unfavorable metastatic patterns and higher rates of visceral metastases, as also observed within our cohort, with a more than doubled rate of M1c disease in mCRPC for radiographically progressing patients, relative to PSA-progressing patients (22% vs. 8.6%).

When patients with conventional staging vs. PSMA-PET/CT at progression to mCRPC were compared, we also made several important observations (Table 3). For example, patients with conventional staging harbored significantly higher rates of high-volume and de novo mHSPC, while rates of local therapy to the prostate were significantly higher in PSMA-PET/CT patients. Moreover, M1a disease in mCRPC was also significantly more frequent in PSMA-PET/CT-staged patients. In univariable cancer-control outcome analyses, PSMA-PET/CT-staged patients outranged conventionally staged mCRPC patients regarding PFS and OS (Figure 2). These observations were also validated in second- and third-line mCRPC and in patients with a PSA level of ≥2 ng/mL (Figure 3). These observations might not only be due to a lead-time bias by earlier diagnoses of castration resistance by PSMA-PET/CT, as when looking at OS with a starting point in mHSPC, significant differences still exist between patients receiving conventional staging and PSMA-PET/CT. However, after adjustments for patient baseline and tumor characteristics, these significant differences in PFS and OS outcomes vanished. These observations indicate that the observed cancer-control outcome advantages may mainly be based on an earlier and more accurate diagnosis of progression or metastasis, which may affect earlier treatment changes and, therefore, lower chances of metastatic progression on unresponsive/resistant ongoing mHSPC/mCRPC treatment. One further explanation might be that a more accurate staging modality with PSMA-PET/CT may also increase the rates of metastasis-directed therapy, which may also affect cancer-control outcomes [25,26]. Last, but not least, patients undergoing PSMA-PET/CT are likely to be treated under more contemporary guidelines and therefore have access to a greater number of and more potent therapy lines. To account for these differences, the multivariable Cox models were adjusted for the year of diagnosis of each patient.

Our study has limitations which should be acknowledged in its interpretation, aside from the retrospective and single-center design. Despite the possible confounding variables adjusted for in the multivariable analyses, potentially further known or unknown variables exist influencing cancer-control outcomes, such as comorbidities other than those considered, genetic profiles, socioeconomic factors or urban vs. rural residence of the patient influencing the availability of staging modalities. However, this is shared by all retrospective studies. Moreover, some analyses may lack an appropriate sample size for further sensitivity analyses, and a potential lead-time bias cannot be completely ruled out for PSMA-PET/CT-staged patients. Therefore, ideally, prospective randomized studies should further elaborate the effect of staging modalities in mCRPC on cancer-control outcomes.

## 5. Conclusions

Initially we hypothesized that patients with radiographic progression towards mCRPC may harbor worse cancer-control outcomes than patients with only biochemical progression due to a rising PSA level. Moreover, we hypothesized that differences in staging modalities may affect cancer-control outcomes. We tested these hypotheses within the FRAMCAP database and conducted the current real-world study which suggests important differences in patients progressing to mCRPC. Specifically, we found that a proportion of over 20% of patients progress without a PSA rise, emphasizing the importance of regular staging intervals. Moreover, we found that patients with worse PSA responses are at higher risk for biochemical progression, emphasizing the need for regular PSA check-ups and kinetic measurements. However, no differences in PFS and OS outcomes between these patients were observed. Finally, staging with PSMA-PET/CT might provide better PFS and OS, relative to conventionally staged mCRPC patients. However, this observation may be substantially influenced by the detection of earlier disease progression and the possibility of treatment in patients with lower metastatic burden, as well as more contemporary patients with better treatment options.

## Figures and Tables

**Table 1:** Characteristics of 356 metastatic castration-resistant prostate cancer (mCRPC) patients stratified according to biochemical (PSA) vs. radiographic vs. both reasons for progression to castration resistance.

Characteristic	N	Overall, N = 356 ^1^	PSA N = 126 (35%) ^1^	Radiographic, N = 81 (23%) ^1^	PSA + Radiographic N = 149 (42%) ^1^	*p*-Value ^2^
**Age mHSPC, years**	344	69 (62, 75)	69 (65, 75)	69 (61, 74)	67 (61, 75)	0.3
**PSA mHSPC, ng/mL**	229	65 (12, 280)	84 (24, 319)	34 (8, 375)	64 (12, 236)	0.2
**PSA nadir mHSPC**	196	0.8 (0.1, 4.0)	1.4 (0.3, 4.1)	0.4 (0.06, 1.1)	0.8 (0.1, 7.7)	0.013
**PSA response ≥ 90% mHSPC**	170	140 (82%)	40 (85%)	37 (88%)	63 (78%)	0.3
**PSA mCRPC, ng/mL**	246	15 (4, 64)	15 (6, 63)	2 (1, 32)	21 (8, 71)	<0.001
**Treatment lines mCRPC**	365	2 (1, 3)	2 (1, 3)	2 (1, 3)	2 (1, 4)	0.029
**ECOG**	262					0.5
0		159 (61%)	54 (54%)	37 (63%)	68 (66%)	
1		90 (34%)	40 (40%)	20 (34%)	30 (29%)	
≥2		13 (5.0%)	6 (6.0%)	2 (3.4%)	5 (4.9%)	
**Cardiovascular disease**	249	85 (34%)	31 (41%)	16 (29%)	38 (32%)	0.3
**Gleason Score**	317					0.3
6–7		91 (29%)	25 (24%)	21 (29%)	45 (33%)	
8–10		226 (71%)	81 (76%)	52 (71%)	93 (67%)	
**Local therapy**	356	131 (37%)	40 (32%)	35 (43%)	56 (38%)	0.2
**De novo mHSPC**	351	230 (66%)	92 (74%)	42 (54%)	96 (64%)	0.012
**High-volume mHSPC**	238	131 (55%)	46 (67%)	30 (51%)	55 (50%)	0.070
**Metastatic sites mCRPC**	218					0.2
M1a		19 (8.7%)	5 (8.6%)	5 (8.6%)	9 (8.8%)	
M1b		171 (78%)	48 (83%)	40 (69%)	83 (81%)	
M1c		28 (13%)	5 (8.6%)	13 (22%)	10 (9.8%)	

^1^ Median (IQR); *n* (%). ^2^ Kruskal–Wallis rank sum test; Fisher’s exact test; Pearson’s Chi-square test. Abbreviations: PSA: prostate-specific antigen, mHSPC: metastatic hormone-sensitive prostate cancer, ECOG: Eastern Cooperative Oncology Group.

**Table 2:** Systemic treatments of 356 metastatic castration-resistant prostate cancer (mCRPC) patients stratified according to biochemical (PSA) vs. radiographic vs. both reasons for progression to castration resistance.

Characteristic	N	Overall, N = 356 ^1^	PSA N = 126 (35%) ^1^	Radiographic, N = 81 (23%) ^1^	PSA + Radiographic N = 149 (42%) ^1^	*p*-Value ^2^
**Treatment mHSPC**	167					0.6
ADT mono		12 (7.2%)	2 (5.0%)	3 (7.1%)	7 (8.2%)	
ARSI		88 (53%)	19 (48%)	24 (57%)	45 (53%)	
Docetaxel		55 (33%)	18 (45%)	11 (26%)	26 (31%)	
Triplet		3 (1.8%)	0 (0%)	0 (0%)	3 (3.5%)	
Other		9 (5.4%)	1 (2.5%)	4 (9.5%)	4 (4.7%)	
**Treatment mCRPC**	356					0.13
ADT mono		31 (8.7%)	18 (14%)	7 (8.6%)	6 (4.0%)	
Chemotherapy		64 (18%)	13 (10%)	20 (25%)	31 (21%)	
Lu-PSMA		18 (5.1%)	3 (2.4%)	7 (8.6%)	8 (5.4%)	
ARSI		176 (49%)	66 (52%)	28 (35%)	82 (55%)	
Radium		15 (4.2%)	7 (5.6%)	3 (3.7%)	5 (3.4%)	
None/Other/NA		52 (15%)	19 (15%)	16 (20%)	17 (11%)	

^1^ Median (IQR); *n* (%). ^2^ Kruskal–Wallis rank sum test; Fisher’s exact test; Pearson’s Chi-square test. Abbreviations: mHSPC: metastatic hormone-sensitive prostate cancer, ADT: androgen deprivation therapy, ARSI: androgen receptor signaling inhibitor, Lu-PSMA: Lutetium Radioligand therapy, NA: unknown.

**Table 3:** Characteristics of 352 metastatic castration-resistant prostate cancer (mCRPC) patients stratified according to staging prior to therapy.

Characteristic	N	Overall N = 352 ^1^	Conventional, N = 235 (67%) ^1^	PSMA, N = 117 (33%) ^1^	*p*-Value ^2^
**Age mHSPC, years**	343	71 (64, 77)	71 (64, 77)	69 (62, 76)	0.15
**PSA mHSPC, ng/mL**	167	62 (13, 298)	75 (13, 417)	54 (12, 105)	0.11
**PSA nadir mHSPC**	159	0.8 (0.1, 3.2)	0.8 (0.1, 4.0)	1.0 (0.3, 2.7)	0.7
**PSA response ≥ 90% mHSPC**	131	112 (85%)	79 (87%)	33 (83%)	0.5
**PSA mCRPC, ng/mL**	259	15 (4, 65)	19 (6, 76)	10 (3, 51)	0.073
**Treatment lines mCRPC**	352	2 (1, 3)	2 (1, 3)	1 (1, 3)	0.2
**ECOG**	274				0.010
0		169 (62%)	105 (56%)	64 (74%)	
1		85 (31%)	64 (34%)	21 (24%)	
≥2		20 (7.3%)	18 (9.6%)	2 (2.3%)	
**Cardiovascular disease**	232	81 (35%)	53 (35%)	28 (34%)	0.9
**Reason for CRPC progression**	204				0.10
PSA		57 (28%)	44 (32%)	13 (19%)	
Radiographic		53 (26%)	31 (23%)	22 (32%)	
Both		94 (46%)	61 (45%)	33 (49%)	
**Gleason Score**	322				0.6
6–7		88 (27%)	57 (26%)	31 (29%)	
8–10		234 (73%)	159 (74%)	75 (71%)	
**Local therapy**	352	159 (45%)	94 (40%)	65 (56%)	<0.01
**De novo mHSPC**	347	195 (56%)	141 (60%)	54 (48%)	0.028
**High-volume mHSPC**	190	107 (56%)	82 (64%)	25 (41%)	<0.01
**Metastatic sites mCRPC**	338				0.002
**M1a**		35 (10%)	14 (6.2%)	21 (19%)	
**M1b**		256 (76%)	178 (79%)	78 (69%)	
**M1c**		47 (14%)	33 (15%)	14 (12%)	

^1^ Median (Q1, Q3); *n* (%). ^2^ Kruskal–Wallis rank sum test; Pearson’s Chi-square test; Fisher’s exact test. Abbreviations: PSA: prostate-specific antigen, mHSPC: metastatic hormone-sensitive prostate cancer, ECOG: Eastern Cooperative Oncology Group.

**Table 4:** Systemic treatment of 352 metastatic castration-resistant prostate cancer (mCRPC) patients stratified according to staging prior to therapy.

Characteristic	N	Overall N = 352 ^1^	Conventional, N = 235 (67%) ^1^	PSMA, N = 117 (33%) ^1^	*p*-Value ^2^
**Treatment mHSPC**	156				<0.01
ADT mono		14 (9.0%)	8 (7.7%)	6 (12%)	
ARSI		82 (53%)	58 (56%)	24 (46%)	
Docetaxel		44 (28%)	34 (33%)	10 (19%)	
Triplet		4 (2.6%)	0 (0%)	4 (7.7%)	
Other		12 (7.7%)	4 (3.8%)	8 (15%)	
**Treatment mCRPC**	352				<0.001
ADT mono		23 (6.5%)	17 (7.2%)	6 (5.1%)	
Chemotherapy		64 (18%)	47 (20%)	17 (15%)	
Lu-PSMA		28 (8.0%)	4 (1.7%)	24 (21%)	
ARSI		162 (46%)	117 (50%)	45 (38%)	
PARPi		2 (0.6%)	0 (0%)	2 (1.7%)	
Radium		16 (4.5%)	11 (4.7%)	5 (4.3%)	
None/Other/NA		57 (16%)	39 (17%)	18 (15%)	

^1^ Median (Q1, Q3); *n* (%). ^2^ Kruskal–Wallis rank sum test; Pearson’s Chi-square test; Fisher’s exact test. Abbreviations: mHSPC: metastatic hormone-sensitive prostate cancer, ADT: androgen deprivation therapy, ARSI: androgen receptor signaling inhibitor, Lu-PSMA: Lutetium Radioligand therapy, PARPi: Poly-(ADP-Ribose) Polymerase Inhibitor, NA: unknown.

## Data Availability

Data are available for bona fide researchers who request them from the authors.
